# Risk factors for Candida colonization and Co-colonization with multi-drug resistant organisms at admission

**DOI:** 10.1186/s13756-015-0089-9

**Published:** 2015-11-14

**Authors:** Danielle M. Schulte, Ajay Sethi, Ronald Gangnon, Megan Duster, Dennis G. Maki, Nasia Safdar

**Affiliations:** Department of Population Health Sciences, University of Wisconsin – Madison School of Medicine and Public Health, Madison, WI USA; Department of Medicine, University of Wisconsin-Madison School of Medicine and Public Health, Madison, WI USA; University of Wisconsin Hospitals and Clinics, Madison, WI USA; William S. Middleton Memorial Veterans Affairs Medical Center, Madison, WI USA

**Keywords:** Candida, Colonization, Multi-drug resistant organisms, Healthcare-associated infections

## Abstract

**Introduction:**

Candida species are major causes of healthcare-associated infections with colonization preceding infection. Understanding risk factors for colonization by Candida species is important in prevention. However, data on risk factors for colonization by Candida species alone or with other healthcare-associated pathogens is limited.

**Methods:**

From 2002 to 2006, 498 patients were enrolled into a prospective cohort study at our institution. Surveillance perirectal, nasal and skin swab samples were obtained upon enrollment. Samples were cultured for the presence of Candida species, Methicillin Resistant *Staphylococcus aureus*, Vancomycin Resistant Enterococcus, and Resistant Gram Negative organisms. Data on demographics, comorbidities, device use, and antibiotic use were also collected for each subject and analyzed using univariate and multivariate logistic regression.

**Results:**

Factors associated with Candida colonization at admission in univariate analysis included ambulatory status, a history of Candida colonization and the use of antibiotics prior to enrollment. In multivariate analysis, ambulatory status (odds ratio; OR = 0.45, 95 % CI: 0.27–0.73) and fluroquinolone use (OR = 3.01, 95 % CI: 1.80–5.01) were associated with Candida colonization at admission. Factors predicting Candida co-colonization with one or more MDROs at admission in univariate analysis included, older age, malnutrition, days spent in an ICU in the 2 years prior to enrollment, a history of MRSA colonization, and using antibiotics prior to enrollment. In multivariate analysis malnutrition (OR = 3.97, 95 % CI: 1.80–8.78) a history of MRSA (OR = 5.51, 95 % CI: 1.89–16.04) and the use of macrolides (OR = 3.75, 95 % CI: 1.18–11.93) and other antibiotics (OR = 4.94, 95 % CI: 1.52–16.03) were associated with Candida co-colonization at admission.

**Discussion:**

Antibiotic use was associated with an increased risk of colonization by Candida species alone and in conjunction with other multidrug-resistant organisms (MDROs). Antibiotic stewardship may be an important intervention for preventing colonization and subsequent infection by Candida and other MDROs.

## Introduction

In the U.S. healthcare-associated infections (HAI) affect about 1.7 million individuals and play a role in approximately 99,000 deaths per year [1, 2]. Candida species are an important healthcare-associated pathogen with bloodstream infections resulting from Candida being the fourth most common cause of hospital acquired bloodstream infection [3]. There is also frequent co-colonization by Candida and other healthcare-associated pathogens [4] which has implications for treatment and can result in various adverse outcomes [5–8].

Colonization precedes infection [9]. Therefore, an understanding of risk factors for colonization is essential to devise effective preventive strategies for infection. However, data on factors predicting colonization by Candida at admission are limited, with most literature focusing on colonization in patients with critical illnesses, long term hospital stays, intensive care unit (ICU) patients or neonates. We undertook an observational cohort study to examine risk factors for rectal colonization by Candida species alone or in conjunction with Methicillin resistant *Staphylococcus aureus* (MRSA), Vancomycin resistant Enterococcus (VRE) and antibiotic resistant Gram-negative organisms (RGN) at admission to our academic facility.

## Methods

### Study setting and protocol

The University of Wisconsin hospital is a 592 bed tertiary care hospital with active solid organ and bone marrow transplant programs. There are 6 ICUs and the patient population comes from all over Wisconsin as well as parts of Illinois and Minnesota. We do not undertake systematic screening for MRSA, VRE or resistant gram negative bacteria. Initiatives to improve hand hygiene have been in place but began after this study had ended. No other specific infection control interventions were in place.

From April 2002 to June 2006, 498 patients were enrolled into a prospective cohort study at the University of Wisconsin hospital. A daily census of hospital admissions was obtained and subjects were randomly selected to be approached for participation. Patients on the psychiatry floor or those in the observation unit (anticipated length of stay < 1 day) were excluded. Consenting patients were enrolled within 1 day of admission to the hospital and were followed until hospital discharge. This study was approved by the institutional review board at our institution.

Surveillance swab samples were obtained from the subject’s nose, rectal area, underarm area, groin, and if applicable, open wound sites upon enrollment and once a week thereafter until discharge. Each rectal area sample was cultured for the presence of Candida, MRSA, VRE, and RGN organisms and the nose, underarm and groin samples were cultured for the presence of MRSA. Data on baseline demographics, comorbidities and device use, and pre- and post-hospital admission antibiotic use were also collected for each subject.

### Laboratory methods

To test for the presence of MRSA, the nasal, underarm, groin, peri-rectal, and wound swabs were inoculated into tryptic soy broth with 6.5% NaCl for 24 h. Fifty microliters were then plated onto Mannitol salt agar plates with 4ug/ml Oxacillin. The plates were then incubated for 48 h and examined for growth. Gram positive cocci were tested for catalase and coagulase for identification of *S. aureus*. All isolates were also tested for resistance to oxacillin using Kirby Bauer Disk Diffusion. To test for the presence of VRE, the rectal swabs were inoculated into bile esculin broth and incubated for 24 h. Fifty microliters were then plated onto bile esculin agar with 6ug/ml vancomycin . Plates were incubated for 48 h and examined for growth. Gram positive cocci were tested for catalase and pyrrolidonyl arylamidase (PYR) for identification of enterococcus. All isolates were tested for resistance to vancomycin by E-test. To test for the presence of RGN organisms, the perirectal swabs were inoculated onto MacConkey agar with 0.5 mg/L of cefotaxime. Gram negative rods were identified using oxidase and analytical profile index (API) testing for enteric and non-enteric bacteria. All isolates were also tested for resistance using Kirby Bauer methods. To test for the presence of Candida, the rectal swabs were inoculated onto Sabouraud’s dextrose agar with chloramphenicol and gentamicin. Each yeast isolate was then identified using API testing for yeast species.

### Definitions

Colonization with Candida was defined as a positive rectal culture for any Candida species. Co-colonization with Candida was defined as a positive rectal culture for any Candida species along with a positive rectal culture for VRE or RGN or a positive rectal, nasal, underarm or groin culture for MRSA. A history of MRSA or Candida was defined as having a previous positive culture with the specified organism.

### Statistical methods

Exploratory data analyses and univariate logistic regression were performed to examine factors associated with Candida colonization alone and with its co-colonization with other MDROs. Factors for which the association with the outcomes of interest were suggestive (p < 0.10) were examined for possible retention in multivariate logistic regression. Parsimonious models were constructed using statistically significant variables and included clinically relevant factors that did not compromise model fit. Unadjusted and adjusted odds ratios and corresponding confidence intervals (CI) were estimated for factors associated with Candida colonization and its co-colonization with MDROs. Subjects with missing data were removed from analysis. Data were analyzed using SAS version 9.4.

## Results

The University of Wisconsin hospital is a 592 bed facility with a patient population that averages 86 % percent white and 5 % African-American 3 % Hispanic/Latino, 1 % Asian and 5 % other. Four-hundred and ninety-eight individuals were enrolled in the study and 8 were ultimately removed from multivariate analysis due to missing data. 275 subjects were male (56 %) and 217 subjects were female (44 %). The average patient age was 56.5 (15.2) years (Table 1). Nineteen percent of subjects were colonized with Candida species at baseline, 4.3 % were colonized with VRE, 1.8 % were colonized with RGN, 1.4 % were colonized with MRSA, and 8.6 % were colonized with multiple organisms.

In univariate analysis, a patient’s need for ventilator support (OR = 3.32, 95 % CI: 1.30–8.50), history of Candida colonization (OR = 2.07, 95 % CI: 1.11–3.84), and use of penicillin combinations (OR = 2.44, 95 % CI: 1.33–4.47) and fluoroquinolone use (OR = 3.25, 95 % CI: 1.98–5.32) prior to admission were significantly associated with higher odds of being colonized by Candida species only, at the time of hospital admission (Table 2). A patient’s ambulatory status (OR = 0.44, 95 % CI: 0.27–0.70) and chemotherapy use (OR = 0.26, 95 % CI: 0.09–0.75) were associated with lower odds of colonization by Candida species.

**Table 1:** Characteristic totals at hospital admission

Characteristic	N	Total with characteristic (SD^Ө^,%)	With Candida (% of total)	Without Candida (% of total)
Age (avg., yr)	491	56.5 (15.2)	58.2 (15.3)	56.0 (15.1)
Body mass index (avg.)	486	28.6 (8.3)	28.3 (8.5)	28.7 (8.3)
Gender (male)	492	275 (55.9 %%)	52 (18.9 %)	223 (81.1 %)
Gender (female)	492	217 (44.1 %)	41 (18.9 %)	176 (81.1 %)
Ambulatory Status (ambulatory)	492	353 (71.7 %)	53 (15.0 %)	300 (85.0 %)
Alcohol use	492	154 (31.3 %)	34 (22.1 %)	120 (77.9 %)
Surgery	492	69 (14.0 %)	7 (10.1 %)	62 (89.9 %)
Coronary artery disease	492	109 (22.2 %)	25 (22.9 %)	84 (77.1 %)
Chemotherapy	492	62 (12.6 %)	4 (6.5 %)	58 (93.5 %)
Lung Disease	492	62 (12.6 %)	13 (21.0 %)	49 (79.0 %)
Hypertension	492	291 (59.1 %)	56 (19.2 %)	235 (80.8 %)
Immunosuppression	492	192 (39.0 %)	35 (18.2 %)	157 (81.8 %)
Malnutrition	492	77 (15.7 %)	20 (26.0 %)	57 (74.0 %)
Cancer	491	152 (31.0 %)	21 (13.8 %)	131 (86.2 %)
Peripheral vascular disease	492	35 (7.1 %)	10 (28.6 %)	25 (71.4 %)
Renal Failure	492	106 (21.5 %)	15 (14.2 %)	91 (85.8 %)
Steroids	492	172 (35.0 %)	29 (16.9 %)	143 (83.1 %)
Abdominal drain	491	21 (4.3 %)	4 (19.0 %)	17 (81.0 %)
Non-insulin dependent diabetes	492	60 (12.2 %)	13 (21.7 %)	37 (61.7 %)
Insulin-dependent diabetes	491	105 (21.4 %)	19 (18.1 %)	86 (81.9 %)
Foley Catheter	492	116 (23.6 %)	24 (20.7 %)	92 (79.3 %)
Hemodialysis	491	29 (5.9 %)	7 (24.1 %)	22 (75.9 %)
Neutropenia	490	14 (2.9 %)	3 (21.4 %)	11 (78.6 %)
Open wound	486	101 (20.8 %)	20 (19.8 %)	81 (80.2 %)
Transplant	490	111 (22.7 %)	21 (18.9 %)	90 (81.1 %)
Vascular catheter	490	462 (94.5 %)	89 (19.3 %)	373 (80.7 %)
Vent support	492	19 (3.9 %)	8 (42.1 %)	11 (57.9 %)
History of Candida	492	56 (11.4 %)	17 (30.4 %)	39 (69.6 %)
History of MRSA^Ө^	492	28 (5.7 %)	5 (17.9 %)	23 (82.1 %)
History of VRE^Ө^	492	8 (1.6 %%)	0 (0 %)	8 (100 %)
History of RGN^Ө^	492	41 (8.3 %)	8 (19.5 %)	33 (80.5 %)
Days in hospital in 2 years. prior (avg,. days)	489	33.8 (108.1)	44.2 (141.6)	31.7(99.6)
Days in ICU in 2 years. prior (avg,. days)	492	1.5 (4.5)	2.1 (4.1)	1.3 (5)
Aminoglycosides^a^	492	11 (2.2 %)	3 (27.3 %)	8 (72.7 %)
Carbapenems^a^	492	4 (0.8 %)	0 (0 %)	4 (100 %)
1st Gen Cephalosporins^a^	492	41 (8.3 %)	5 (12.2 %)	36 (87.8 %)
2nd Gen Cephalosporins^a^	492	10 (2.0 %)	1 (10.0 %)	9 (90.0 %)
3rd Gen Cephalosporins^a^	492	34 (6.9 %)	7 (20.6 %)	27 (79.4 %)
4th Gen Cephalosporins^a^	492	12 (2.4 %)	2 (16.7 %)	10 (83.3 %)
Glycopeptides^a^	492	32 (6.5 %)	7 (21.9 %)	25 (78.1 %)
Lincosamides^a^	492	23 (4.7 %)	7 (30.4 %)	16 (69.6 %)
Macrolides^a^	492	29 (5.9 %)	6 (20.7 %)	23 (79.3 %)
Nitrofurans^a^	492	5 (1.0 %)	1 (20.0 %)	4 (80.0 %)
Penicillins^a^	492	26 (5.3 %)	5 (19.2 %)	21(80.8 %)
Penicillin Combinations^a^	492	57 (11.6 %)	19 (33.3 %)	38 (66.7 %)
Fluoroquinolones^a^	492	101 (20.5 %)	36 (35.6 %)	65 (64.4 %)
Sulfonamides^a^	492	62 (12.6 %)	12 (19.4 %)	50 (80.6 %)
Tetracyclines^a^	492	3 (0.6 %)	2 (66.7 %)	1 (33.3 %)
Drugs against mycobacteria^a^	492	5 (1.0 %)	1 (20.0 %)	4 (80.0 %)
Other antibiotics^b^	492	21 (4.3 %)	6 (28.6 %)	15 (71.4 %)

^Ө^ *SD* standard deviation, *MRSA* Methicillin resistant *Staphylococcus* aureus, *VRE* Vancomycin resistant Enterococcus, *RGN* antibiotic resistant Gram-negative organisms

All variables are dichotomous, yes vs. no, unless otherwise stated

^a^Antibiotic use occurred prior to admission to hospital

^b^Other antibiotics included use of either Metronidazole, Dapsone or Linezolid

**Table 2:** Univariate odds ratios of Candida colonization at hospital admission

Characteristic	Odds ratio	95 % Confidence interval
Age (yr)	1.01	0.99–1.03
Body mass index	0.99	0.97 – 1.02
Gender (male vs. female)	1.00	0.63–1.57
Ambulatory Status (ambulatory vs non-ambulatory)	0.44	0.27 – 0.70
Alcohol use	1.34	0.84–2.15
Surgery	0.44	0.20–1.00
Coronary artery disease	1.38	0.82–2.31
Chemotherapy	0.26	0.09–0.75
Lung Disease	1.16	0.60–2.24
Hypertension	1.06	0.67–1.67
Immunosuppression	0.93	0.58–1.48
Malnutrition	1.67	0.94 – 2.95
Cancer	0.59	0.35–1.01
Peripheral vascular disease	1.80	0.83–3.90
Renal Failure	0.65	0.36–1.19
Steroids	0.81	0.50–1.32
Abdominal drain	1.01	0.33–3.07
Non-insulin dependent diabetes	1.59	0.81–3.13
Insulin-dependent diabetes	0.93	0.53–1.63
Foley Catheter	1.16	0.69–1.95
Hemodialysis	1.39	0.57 – 3.35
Neutropenia	1.17	0.32–4.28
Open wound	1.09	0.63–1.90
Transplant	1.00	0.58–1.71
Vascular catheter	1.37	0.46 – 4.07
Vent support	3.32	1.30–8.50
History of Candida	2.07	1.11–3.84
History of MRSA^Ө^	0.93	0.34–2.51
History of VRE^Ө^	<0.001	<0.001– >999.99
History of RGN^Ө^	1.04	0.47–2.34
Days in hospital in 2 years. prior^†^ (days)	1.00	1.00–1.00
Days in ICU in 2 years. prior^†^ (days)	1.03	0.99–1.07
Aminoglycosides^a^	1.63	0.42– 6.26
Carbapenems^a^	<0.001	<0.001– >999.99
1st Gen Cephalosporins^a^	0.57	0.22–1.50
2nd Gen Cephalosporins^a^	0.47	0.06–3.76
3rd Gen Cephalosporins^a^	1.12	0.47–2.66
4th Gen Cephalosporins^a^	0.86	0.18–3.97
Glycopeptides^a^	1.22	0.51–2.91
Lincosamides^a^	1.95	0.78–4.88
Macrolides^a^	1.13	0.45–2.85
Nitrofurans^a^	1.08	0.12–9.72
Penicillins^a^	1.02	0.38–2.79
Penicillin Combinations^a^	2.44	1.33–4.47
Fluoroquinolones^a^	3.25	1.98–5.32
Sulfonamides^a^	1.03	0.53–2.03
Tetracyclines^a^	8.75	0.79–97.51
Drugs against mycobacteria^a^	1.08	0.12–9.72
Other antibiotics^b^	1.77	0.67–4.68

^Ө^MRSA = Methicillin resistant *Staphylococcus* aureus; VRE = Vancomycin resistant Enterococcus; RGN = antibiotic resistant Gram-negative organisms

All variables are dichotomous, yes vs. no, unless otherwise stated

^†^Estimate for each additional day in the hospital/ intensive care unit

^a^Antibiotic use occurred prior to admission to hospital

^b^Other antibiotics included use of either Metronidazole, Dapsone or Linezolid

In multivariate analysis, adjusting for age, and other factors included in the model patients who could ambulate had 55 % lower odds of being colonized with only Candida at admission (OR = 0.45, 95 % CI: 0.27–0.73). Patients who used quinolones prior to hospital admission had three times greater odds of being colonized with Candida at admission to the hospital (OR = 3.01, 95 % CI: 1.80–5.014) (Table 3).

**Table 3:** Multivariate odds ratios of Candida colonization at hospital admission (n = 485)

Characteristic	Multivariate odds ratio	95 % Confidence interval	Univariate odds ratio	95 % Confidence interval
Body mass index	0.99	0.96–1.02	0.99	0.97 – 1.02
Age (yr)	1.01	0.99–1.02	1.01	0.99–1.03
Ambulatory Status (ambulatory vs non-ambulatory)	0.45	0.27–0.73	0.44	0.27 – 0.70
History of Candida	1.75	0.91–3.37	2.07	1.11–3.84
Fluoroquinolones^a^	3.01	1.80–5.01	3.25	1.98–5.32

^a^Antibiotic use occurred prior to admission to hospital

**Table 4:** Characteristic totals for co-colonization at hospital admission

Characteristic	N	Total (SD^Ө^,%)	With Co-col (SD^Ө^, %)	Without Co-col (SD^Ө^, %)
Age (avg., yr)	491	56.4 (15.1)	58.9 (17.4)	56.3 (15.0)
Body mass index (avg.)	486	28.6 (8.3)	28.9 (9.2)	28.6 (8.2)
Gender (male)	492	275 (55.9 %)	15 (5.8 %)	260 (94.5 %)
Gender (female)	492	217 (44.1 %)	18 (8.3 %)	199 (91.7 %)
Ambulatory Status (ambulatory)	492	353 (71.7 %)	21(5.9 %)	332 (94.1 %)
Alcohol use	492	154 (31.3 %)	12 (7.8 %)	142 (92.2 %)
Surgery	492	69 (14.0 %)	2 (2.9 %)	67 (97.1 %)
Coronary artery disease	492	109 (22.2 %)	8 (7.3 %)	101 (92.7 %)
Chemotherapy	492	62 (12.6 %)	3 (4.8 %)	59 (95.2 %)
Lung Disease	492	62 (12.6 %)	6 (9.7 %)	56 (90.3 %)
Hypertension	492	291 (59.1 %)	18 (6.2 %)	273 (93.8 %)
Immunosuppression	492	192 (39.0 %)	10 (5.2 %)	182 (94.8 %)
Malnutrition	491	77 (15.7 %)	14 (18.2 %)	63 (81.8 %)
Cancer	491	152 (31.0 %)	9 (5.9 %)	143 (94.1 %)
Peripheral vascular disease	492	35 (7.1 %)	3 (8.6 %)	32 (91.4 %)
Renal Failure	492	106 (21.2 %)	11 (10.4 %)	95 (89.6 %)
Steroids	492	172 (35.0 %)	9 (5.2 %)	163 (94.8 %)
Abdominal drain	491	21 (4.3 %)	1 (4.8 %)	20 (95.2 %)
Non-insulin dependent diabetes	492	50 (10.2 %)	4 (8.0 %)	46 (92.0 %)
Insulin-dependent diabetes	491	105 (21.4 %)	8 (7.6 %)	97 (92.4 %)
Foley Catheter	492	116 (24.2 %)	12 (10.3 %)	104 (89.7 %)
Hemodialysis	490	29 (5.9 %)	3 (10.3 %)	26 (89.7 %)
Neutropenia	490	14 (2.9 %)	1 (7.1 %)	13 (92.9 %)
Open wound	486	101 (20.8 %)	10 (9.9 %)	91 (90.1 %)
Surgical wound	490	76 (15.5 %)	6 (7.9 %)	70 (92.1 %)
Transplant	490	111 (22.7 %)	4 (3.6 %)	107 (96.4 %)
Vascular catheter	489	462 (94.5 %)	31 (6.7 %)	431 (93.3 %)
Vent support	492	19 (3.9 %)	2 (10.5 %)	17 (89.5 %)
History of Candida	492	56 (11.4 %)	5 (8.9 %)	51 (91.1 %)
History of MRSA^Ө^	492	28 (5.7 %)	8 (28.6 %)	20 (71.4 %)
History of VRE^Ө^	492	8 (1.6 %)	1 (12.5 %)	7 (87.5 %)
History of GN^Ө^	492	41 (8.3 %)	4 (9.8 %)	37 (90.2 %)
Days in hospital in 2 years. prior to admit (avg., days)	489	34.1 (108.8)	34.1 (40.5)	34.1 (112.1)
Days in ICU in 2 years. prior to admit (avg., days)	492	1.5 (4.9)	4.5 (9.6)	1.3 (4.3)
Aminoglycosides^a^	492	11 (2.2 %)	1 (9.1 %)	10 (90.9 %)
Carbapenems^a^	492	4 (0.8 %)	2 (50.0 %)	2 (50.0 %)
1st Gen Cephalosporins^a^	492	41 (8.3 %)	3 (7.3 %)	38 (92.7 %)
3rd Gen Cephalosporins^a^	492	34 (6.9 %)	5 (14.7 %)	29 (85.3 %)
4th Gen Caphalosporins^a^	492	12 (2.4 %)	1 (8.3 %)	11 (91.7 %)
Glycopeptides	492	32 (6.5 %)	5 (15.6 %)	27 (84.4 %)
Lincosamides^a^	492	23 (4.7 %)	1 (4.3 %)	22 (95.7 %)
Macrolides^a^	492	29 (5.9 %)	5 (17.2 %)	24 (82.8 %)
Penicillins^a^	492	26 (5.3 %)	4 (15.4 %)	22 (84.6 %)
Penicillin Combinations^a^	492	57 (11.6 %)	5 (8.8 %)	52 (91.2)
Fluoroquinolones^a^	492	101 (20.5 %)	10 (9.9 %)	91 (90.1 %)
Sulfonamides^a^	492	62 (12.6 %)	1 (1.6 %)	61 (98.4 %)
Drugs against mycobacteria^a^	492	5 (1.0 %)	2 (40.0 %)	3 (60.0 %)
Other antibiotics^b^	492	21 (4.3 %)	5 (23.8 %)	16 (76.2 %)

^Ө^ *SD* standard deviation, *MRSA* Methicillin resistant *Staphylococcus* aureus, *VRE* Vancomycin resistant Enterococcus, *RGN* antibiotic resistant Gram-negative organisms

All variables are dichotomous, yes vs. no, unless otherwise stated

^a^Antibiotic use occurred prior to admission to hospital

^b^Other antibiotics included use of either Metronidazole, Dapsone or Linezolid

We examined factors for co-colonization by Candida species and one or more of the other organisms of interest in this study (Table 4). In univariate analysis, malnutrition (OR = 4.62, 95 % CI: 2.21–9.68), history of MRSA colonization (OR = 7.03, 95 % CI: 2.82–17.52), days spent in an ICU in the two years prior to their present hospital admission (OR = 1.07, 95 % CI: 1.03–1.12), and use of carbapenems (OR = 14.74, 95 % CI: 2.01–108.22), glycopeptides (OR = 2.86, 95 % CI: 1.02–7.99), macrolides (OR = 3.24, 95 % CI: 1.15–9.13), drugs against Mycobacteria (OR = 9.81, 95 % CI: 1.58–60.88) and either metronidazole, dapsone, or linezolid (OR = 4.94, 95 % CI: 1.69–14.48) were significantly associated with a higher odds of being co-colonized with Candida and another organism at the time of hospital admission (Table 5).

**Table 5:** Univariate odds ratios of Candida co-colonization at hospital admission

Characteristic	Odds ratio	95 % Confidence interval
Age (yr)	1.01	0.99–1.04
Body mass index	1.01	0.97 – 1.05
Gender (male vs. female)	1.59	0.77–3.19
Ambulatory Status (ambulatory vs. non-ambulatory)	0.67	0.32 – 1.40
Alcohol use	1.28	0.61–2.66
Surgery	0.38	0.09–1.62
Coronary artery disease	1.13	0.50–2.59
Chemotherapy	0.68	0.20–2.29
Lung Disease	1.60	0.63–4.04
Hypertension	0.82	0.40–1.66
Immunosuppression	0.66	0.31–1.42
Malnutrition	4.62	2.21–9.68
Cancer	0.83	0.37–1.82
Peripheral vascular disease	1.34	0.39–4.61
Renal Failure	1.92	0.90–4.09
Steroids	0.68	0.31–1.50
Abdominal drain	0.68	0.09–5.26
Non-insulin dependent diabetes	1.24	0.42–3.68
Insulin-dependent diabetes	1.19	0.52–2.72
Foley Catheter	1.95	0.93–4.10
Hemodialysis	1.72	0.49 – 6.02
Neutropenia	1.07	0.14 – 8.42
Open wound	1.73	0.80–3.76
Surgical wound	1.23	0.49 – 3.09
Transplant	0.47	0.16–1.37
Vascular catheter	0.90	0.20 – 3.97
Vent support	1.68	0.37–7.59
History of Candida	1.43	0.53–3.86
History of MRSA^Ө^	7.03	2.82 – 17.52
History of VRE^Ө^	2.02	0.24 – 16.91
History of GN^Ө^	1.57	0.53–4.72
Days in hospital in 2 years. prior to admit^†^ (days)	1.00	1.00–1.00
Days in ICU in 2 years. prior to admit^†^ (days)	1.07	1.03–1.12
Aminoglycosides^a^	1.41	0.17–11.31
Carbapenems^a^	14.74	2.01–108.22
1st Gen Cephalosporins^a^	1.11	0.32–3.80
3rd Gen Cephalosporins^a^	2.65	0.95–7.37
4th Gen Caphalosporins^a^	1.27	0.16 – 10.17
Glycopeptides	2.86	1.02 – 7.99
Lincosamides^a^	0.62	0.08–4.75
Macrolides^a^	3.24	1.15–9.13
Penicillins^a^	2.74	0.89–8.48
Penicillin Combinations^a^	1.40	0.52–3.78
Fluoroquinolones^a^	1.76	0.81–3.82
Sulfonamides^a^	0.20	0.03–1.52
Drugs against mycobacteria^a^	9.81	1.58 – 60.88
Other antibiotics^b^	4.94	1.69–14.48

^Ө^ *MRSA* Methicillin resistant *Staphylococcus* aureus, *VRE* Vancomycin resistant Enterococcus, *RGN* antibiotic resistant Gram-negative organisms All variables are dichotomous, yes vs. no, unless otherwise stated

^†^Estimate for each additional day in the hospital/ intensive care unit

^a^Antibiotic use occurred prior to admission to hospital

^b^Other antibiotics included use of either Metronidazole, Dapsone or Linezolid

In multivariate analysis, adjusting for age, and other factors included in the model, we found that, at admission, malnourished individuals had a four-fold greater odds of being co-colonized (OR = 3.97, 95 % CI: 1.80–8.78) and individuals with a history of MRSA had over a five-fold greater odds of being co-colonized (OR = 5.51, 95 % CI: 1.89–16.04). Individuals who used macrolides prior to admission had almost a four-fold greater odds of being co-colonized (OR = 3.75, 95 % CI: 1.18–11.93) and individuals who used either metronidazole, dapsone, or linezolid had a five-fold greater odds of being co-colonized (OR = 4.94, 95 % CI: 1.52–16.03) (Table 6).

**Table 6:** Multivariate odds ratios of Candida co-colonization at hospital admission (n = 490)

Characteristic	Multivariate Odds ratio	95 % Confidence limits	Univariate Odds ratio	95 % Confidence limits
Age (yr)	1.03	1.01–1.06	1.01	0.99 - 1.04
Malnutrition	3.97	1.80–8.78	4.62	2.21–9.68
History of MRSA	5.51	1.89–16.04	7.03	2.82 – 17.52
Days in ICU in 2 years prior to admission^†^ (days)	1.05	1.00–1.11	1.07	1.03–1.12
Macrolides^a^	3.75	1.18v11.93	3.24	1.15–9.13
Other antibiotics^b^	4.94	1.52–16.03	4.94	1.69–14.48

^†^Estimate for each additional day in the intensive care unit

^a^Antibiotic use occurred prior to admission to hospital

^b^Other antibiotics included use of either Metronidazole, Dapsone or Linezolid

## Discussion

Infection by Candida species in hospitalized patients is associated with considerable morbidity and mortality. Prevention is essential but a better understanding of the factors that predict colonization by Candida species is needed. Our study expands the current literature in this area. We identified a number of factors that predicted colonization by Candida species alone or in conjunction with other MDROs. Specifically, we found that individuals who were able to ambulate were less likely to be colonized with Candida species at enrollment and those using Fluoroquinolones prior to admission were more likely to be colonized with Candida species at enrollment. Additionally, we identified several factors that predicted co-colonization with other MDROs at enrollment. These included malnutrition, a history of MRSA, and the use of Macrolides or the use of either Metronidazole, Dapsone, or Linezolid prior to hospital admission.

We found that subjects who could ambulate as opposed to those who could not were about 50 % less likely to be colonized with Candida at enrollment. These individuals were more likely to have been mobile, and not been confined to a hospital or long-term care facility. Our findings are consistent with studies that have shown that individuals who can ambulate are less likely to develop infections [10–13], one study even suggested that patients who are admitted to long-term care facilities ambulate more frequently as a form of infection prevention [14].

We also found that subjects with a history of Candida colonization were more likely to be colonized with Candida at admission than those without this history. Our findings are in keeping with previous studies which reported that colonization with Candida species may be prolonged [15].

Additionally we identified factors that were associated with an increase in the risk of co-colonization by Candida and at least one other MDRO. Subjects with malnutrition showed an increased risk of being co-colonized at baseline. Malnutrition can impair an individual’s immunity and cause immune dysfunction, thus affecting the body’s ability to fight infection [16, 17]. Accordingly, this impairment of an individual’s immune system as a result of their malnutrition could be the driving force associating malnutrition and a higher likelihood of co-colonization.

Having a history of MRSA colonization was also indicative of being co-colonized at baseline. Previous studies have shown an association between having a history of MRSA and having a positive MRSA screening sample at hospital admission [18] as well as an association between having a history of *Staphylococcus* colonization and developing a surgical site infection [19]. This suggests then that having a history of MRSA could be a predictor of baseline colonization with not only MRSA but other organisms as well, including Candida. Colonization with MRSA can also be indicative of a previous hospitalization or previous use of antibiotics; these variables have been shown to be associated with colonization and therefore may also be mediating this relationship [20].

The use of certain antibiotics was implicated with a higher likelihood of colonization and co-colonization. Previous studies have also highlighted the association existing between antibiotic use and Candida colonization [21–25]. It has been found that the use of antibiotics with broad-spectrum activity were associated with higher levels of *C. albicans* colonization and accordingly our study also found that subjects taking certain antibiotics prior to admission were more likely to be colonized and co-colonized at enrollment [21]. More specifically, we have observed that the use of quinolones prior to hospitalization was associated with an increase in a subject’s likelihood of being colonized with Candida at baseline and the use of macrolides and either metronidazole, dapsone or linezolid prior to hospitalization was associated with an increase in a subject’s likelihood of being co-colonized with Candida and another organism at baseline. It has been suggested that individuals taking antibiotics were more likely have a reduction in intestinal bacteria, allowing Candida to opportunistically grow, thus resulting in an increase in intestinal Candida counts [22] which would support this observed association. Information on the the duration of antibiotic exposure and whether or not antibiotics were appropriately utilized by each subject was unavailable in this study but would be an important point of interest for future studies. In this regard however, our study has highlighted the possible antibiotics that should be more closely monitored in future studies and in a clinical setting when prescribed to patients.

A limitation of this study was its small sample size, which will restrict the power of our associations. We also lacked information on length of use and date of last dose for pre-admission antibiotics, which limited our ability to fully describe the relationship between these drugs and colonization. Additionally, we repeated surveillance testing no more frequently than every 3 days, so a positive test after the admission test was negative was a priori defined as acquisition. It is possible that our admission testing resulted in false negatives, however we followed procedure similar to that of most studies that have evaluated acquisition. Finally, we lacked information on possible pre- and post-admission antifungal use by the subjects in our dataset and cannot make any assertions about the role these drugs might play on the outcome of colonization and co-colonization. The use of antifungals however is an important factor to analyze in future studies.

## Conclusion

Our study has shown that ambulatory status, malnutrition, a history of MRSA colonization, and antibiotic use prior to admission were significant risk factors for Candida colonization and co-colonization at hospital admission. These risk factors are important indicators of colonization to be examined in patients at hospital admission so as to better anticipate and prevent colonization and co-colonization with Candida species and MRSA, VRE and RGN organisms. Also, given that antibiotic use is a directly mutable variable, interventions should be directed at promoting appropriate antibiotic administration and utilization. More research should now be performed to more completely understand the mechanisms by which these risk factors can affect an individual’s colonization status so as to improve prediction and prevention procedures for colonization and ultimately protect patients from avoidable infection.

## Abbreviations

API: Analytical profile index
CI: Confidence intervals
MDROs: Multi-drug resistant organisms
MRSA: Methicillin resistant *Staphylococcus aureus*
OR: Odds Ratio
RGN: Antibiotic resistant Gram negative organisms
VRE: Vancomycin resistant Enterococcus
